# New cassava germplasm for food and nutritional security in Central Africa

**DOI:** 10.1038/s41598-021-86958-w

**Published:** 2021-04-01

**Authors:** Isaac Tize, Apollin Kuate Fotso, Elias Nchiwan Nukenine, Cargele Masso, Francis Ajebesone Ngome, Christopher Suh, Venasius Wirnkar Lendzemo, Ibrahim Nchoutnji, Gabriel Manga, Elisabeth Parkes, Peter Kulakow, Christiant Kouebou, Komi K. M. Fiaboe, Rachid Hanna

**Affiliations:** 1 International Institute of Tropical Agriculture, BP 2008, Messa, Yaoundé, Cameroon; 2 Department of Biological Sciences, University of Ngaoundere, BP 454, Ngaoundere, Cameroon; 3 Institute of Agricultural Research for Development, BP 2067, Yaoundé, Cameroon; 4 International Institute of Tropical Agriculture, PMB 320 Oyo Road, Ibadan, Nigeria; 5 Agricultural Investment and Market Development Project, Yaoundé, Cameroon; 6 Present Address: Congo Basin Institute, Institute of Environment and Sustainability, University of California, Box 951496, Los Angeles, USA

**Keywords:** Plant breeding, Malnutrition

## Abstract

Cassava is a key food security crop in Central Africa, but its production depends largely on the use of local farmers’ varieties characterized by inherently low yield which is compounded by generally high susceptibility to various growth and yield-limiting pests and diseases. Improved cassava genotypes have demonstrated the potential to substantially improve cassava’s contribution to food security and the development of the cassava industry and the improvement of nutrition status elsewhere in Western Africa. Eleven improved cassava genotypes were compared with a local landrace (LMR) used as a check under field conditions over two years in eight locations, grouped in four agro-ecologies in Cameroon. Pest and disease abundance/incidence and damage severity were evaluated. At harvest, root yield and carotenoid content were measured. Best linear unbiased predictors showed the lowest breeding value for LMR with the cassava mosaic virus disease (+ 66.40 ± 2.42) compared with 1.00 ± 0.02% for the most susceptible improved genotype. Two genotypes (I010040-27 and I011797) stood out for having higher predicted fresh root yield means which were at least 16 times greater compared with LMR. Predicted total carotenoid content was the highest (+ 5.04 ± 0.17) for improved genotype I070593 compared with LMR which showed the lowest (− 3.90 ± 0.06%) and could contribute to the alleviation of vitamin A deficiency from cassava-based food systems. Diffusion of high-yielding and nutritious genotypes could alleviate food and nutritional security in Central Africa.

## Introduction

Cassava (*Manihot esculenta* Crantz), is the main source of calories for 800 million people across the globe^1^. No other continent depends on cassava to feed as many people as does Africa^2^. Its production is estimated at 178 million tons—61% of global production—produced annually in sub-Saharan Africa (SSA)^3^. Population growth and demands by emerging cassava industries are increasingly straining current production capacities that rely widely on low-yielding farmers’ local varieties that are particularly susceptible to widespread and emerging pests and diseases such as cassava mosaic virus disease, cassava brown streak virus disease, and cassava whiteflies^4–8^. Moreover, the local varieties are deficient in micronutrients, thus their consumption could lead to micronutrient deficiency, also referred to as hidden hunger^9^. Acute deficiencies in one or more micronutrients, including vitamins A, C, E, and minerals are reported in about two billion people^9^. In Cameroon, about 39% of preschool-aged children and 18% of pregnant women are deficient in vitamin A. For example, acute malnutrition is estimated at around 4.8% in the East region of Cameroon^10^. According to UNHCR (2018), nearly 265,000 refugees from the Central African Republic have sought shelter in eastern Cameroon, increasing the level of food insecurity in the region.

Aiming at improving the production of cassava on the continent, the International Institute of Tropical Agriculture (IITA) and its partners have developed new cassava genotypes to improve the livelihoods of millions of farmers in SSA. It is now widely recognized that traditional staple crops (rice, wheat, maize, and soybean) are no longer providing adequate and enough solutions to the world’s food insecurity^11–14^. Cassava in this regard is recognized as a crop that can contribute to global food security^12,15–18^.

Cassava is resistant to adverse environments^19^; however, its production in Cameroon and elsewhere—particularly in Central Africa—is constrained by heavy yield losses from pests and diseases among which are: (1) cassava mosaic virus disease (CMD) transmitted largely by the whitefly *Bemisia tabaci* (G.) and by infected cassava stems used as vegetative propagules; (2) cassava anthracnose disease (CAD) caused by the fungus *Colletotrichum gloeosporioides* (Penz.) Sacc. and also vectored by *Pseudotheraptus devastans* Dist. (Het. Coreidae); and (3) cassava green mite (CGM) *Mononychellus tanajoa* (B.)^20–23^. CMD is the most challenging disease as it can cause 25–90% yield losses, with substantial negative impact on people’s livelihoods^5,6,24^, particularly where CMD-susceptible traditional farmers cassava varieties are used compared with the CMD-resistant improved varieties^4,8^. Host plant resistance provides the cheapest and simplest technology where chemical inputs are not available or affordable.

Crop improvement has traditionally focused on increasing crop yield and building resistance to pests and diseases. In recent years, enhancement of crops nutrient has been increasingly incorporated in breeding programs. Biofortification of staple food crops like cassava has been advocated as one of the cost-effective solutions to combat the scourge of micronutrient malnutrition^25^ and for sustainable improvement in the lives of millions of people in developing countries, particularly in Africa and South Asia^25–27^.

Genotype x environment interaction is the greatest challenge to breeders due to differential genotypic responses across environments^15,28–32^. In a previous study^8^, we evaluated a set of 18 cassava genotypes, mostly oriented toward industrial processing, across eight environments in Cameroon to assess the level of stability of their yield and pest and disease resistance. The principal objective of the present study was to evaluate and select new cassava genotypes suitable for non-fermented products prepared using a single-step process after peeling (boiling or frying) and with high resistance to CMD and other pests, and with higher provitamin A content under specific environments. Broadly, the results from this Cameroon study are likely to be extended to countries sharing similar ecologies, mainly in the Congo Basin, as Cameroon, with its varied environments, is considered the gateway to the rest of Central Africa.

## Results

### Soil nutrient analysis

The levels of soil pH and various soil chemical characteristics varied among locations. Soil pH in Foumbot (6.06 ± 0.02) differed significantly from the other locations, with Bambui and Njombe being most acidic (3.93 ± 0.01 and 3.93 ± 0.06 respectively). The highest organic carbon content (6.96 ± 0.03) was recorded in Bambui. Ekona, Foumbot, and Meiganga differed from the other locations with their higher level of Ca, Mg, and P. Meyomessala soil had the highest sand content while Njombe and Bambui soils had the highest clay and silt respectively (Table 1).

**Table 1:** Average soil physical and chemical characteristics (± SE).

Locations	pH	Ca	Mg	K	P	Org C	Total N	Sand	Clay	Silt
	Water	cmol( +)/kg			ug/g	%				
Bambui	3.93 ± 0.01^d^	0.97 ± 0.05^f^	0.37 ± 0.02^f^	0.26 ± 0.01^d^	2.44 ± 0.05^d^	6.96 ± 0.03^a^	0.44 ± 0.00^a^	54.2 ± 0.39^c^	18.3 ± 0.36^e^	27.5 ± 0.14^a^
Gamboula	4.11 ± 0.03^d^	1.21 ± 0.08^ef^	0.74 ± 0.04^e^	0.14 ± 0.00^d^	8.10 ± 0.4^d^	2.32 ± 0.04^e^	0.14 ± 0.00^ fg^	54.3 ± 0.41^c^	36.6 ± 0.45^b^	9.17 ± 0.25^e^
Ekona	4.91 ± 0.05^bc^	9.64 ± 0.23^c^	3.94 ± 0.08^b^	1.77 ± 0.07^a^	61.3 ± 2.67^a^	3.50 ± 0.04^d^	0.35 ± 0.00^c^	48.6 ± 0.69^d^	27.3 ± 0.54^c^	24.1 ± 0.41^c^
Foumbot	6.06 ± 0.02^a^	15.4 ± 0.18^a^	5.21 ± 0.12^a^	1.54 ± 0.05^b^	20.9 ± 0.7^bc^	5.74 ± 0.03^b^	0.40 ± 0.01^b^	63.6 ± 0.36^b^	11.2 ± 0.34f.	25.2 ± 0.23^b^
Mbalmayo	4.70 ± 0.05^c^	3.01 ± 0.21^def^	1.27 ± 0.07^d^	0.16 ± 0.01^d^	10.1 ± 1.7^ cd^	1.73 ± 0.2f.	0.15 ± 0.00f.	61.30 ± 0.85^b^	26.0 ± 0.80^ cd^	12.7 ± 0.22^d^
Meiganga	5.32 ± 0.08^b^	11.9 ± 1.07^b^	2.51 ± 0.06^c^	0.55 ± 0.02^c^	71.4 ± 6.6^a^	5.15 ± 0.05^c^	0.30 ± 0.00^d^	53.9 ± 0.68^c^	23.5 ± 0.41^d^	22.7 ± 0.37^bc^
Meyomessala	4.84 ± 0.04^c^	3.77 ± 0.57^d^	0.90 ± 0.11^e^	0.18 ± 0.02^d^	29.2 ± 2.3^b^	1.65 ± 0.05f.	0.12 ± 0.00^ g^	81.7 ± 0.53^a^	12.1 ± 0.42^f^	6.23 ± 0.24^f^
Njombe	3.93 ± 0.06^d^	2.50 ± 0.33^de^	0.88 ± 0.10^de^	0.19 ± 0.03^d^	8.75 ± 1.7^ cd^	2.33 ± 0.15^e^	0.18 ± 0.01^e^	45.6 ± 2.23^d^	43.7 ± 2.90^a^	10.6 ± 1.41^e^
Mean	4.72	6.06	1.98	0.60	26.5	3.67	0.26	57.9	24.8	17.3
SE	0.26	1.94	0.62	0.24	9.23	0.72	0.05	3.99	4.00	2.98
CV	15.6	90.8	88.7	111.4	98.4	55.3	49.7	19.5	45.6	48.8

Means in a column followed by the same letter are not significantly different.

*SE* standard error, *CV* coefficient of variation.

### Best linear unbiased predictors (BLUPs) for the diseases: CMD and CAD

**Figure 1:**
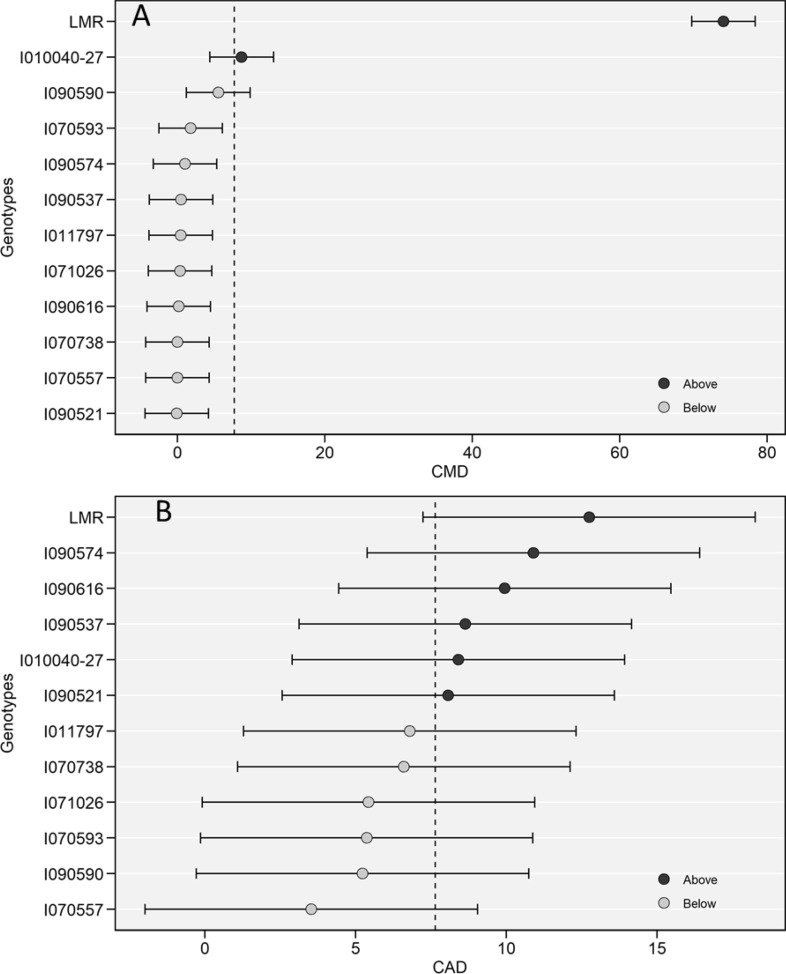
Best linear unbiased prediction of CMD (**A**) and CAD (**B**) incidence (%) for 12 cassava genotypes. Black and grey circles represent the genotypes that had BLUP above and below of BLUP mean, respectively. Horizontal error bars represent the 95% confidence interval of a prediction considering a 2-tailed *t* test. (R V3.6.2, https://cran.r-project.org/web/packages/metan/).

The predicted breeding values of CMD ranged from − 7.79 ± 2.42 for genotype I090521, which did not show any disease symptoms, to + 66.40 ± 2.42 for the local landrace LMR, which had the highest CMD incidence and severity (+ 1.36 ± 0.10) (Table 2). The most infected improved variety was I010040-27 with a BLUP of + 1.00 ± 0.02%. Other genotypes performed below the grand mean and with a very small difference among them (Fig. 1A).

**Table 2:** Best linear unbiased predictors (BLUPs) of breeding values with standard errors for cassava mosaic disease (CMD) incidence (%) and severity, cassava anthracnose disease (CAD) incidence (%), and densities of, whiteflies (WF), cassava green mite (CGM).

Genotype/statistics	CMD incidence	CMD severity	CAD incidence	CAD severity	WF	CGM
I010040-27	+ 1.00 ± 0.02	+ 0.24 ± 0.25	+ 0.50 ± 3.61	+ 0.06 ± 0.01	− 7.30 ± 7.39	+ 1.26 ± 4.79
I070557	− 7.72 ± 2.42	− 0.23 ± 0.27	− 4.43 ± 2.61	− 0.07 ± 0.15	+ 9.13 ± 5.32*	− 0.25 ± 3.45
I011797	− 7.28 ± 19.8	− 0.18 ± 1.44	− 1.12 ± 2.48	+ 0.01 ± 0.00	− 2.28 ± 6.67	− 2.28 ± 4.33
I090590	− 2.17 ± 2.42	+ 0.11 ± 0.16	− 2.71 ± 3.61	+ 0.00 ± 0.00	− 7.6 ± 6.58	− 0.73 ± 4.27
I090537	− 7.23 ± 2.42	− 0.07 ± 0.38	+ 0.71 ± 3.61	+ 0.03 ± 0.08	− 2.74 ± 5.62	− 0.94 ± 3.64
I070738	− 7.72 ± 2.42	− 0.22 ± 0.42*	− 1.62 ± 3.61	− 0.03 ± 0.10	− 7.18 ± 6.53	− 2.85 ± 4.23
I090574	− 6.67 ± 2.42	− 0.09 ± 0.60	+ 3.03 ± 2.61	+ 0.03 ± 0.0	+ 9.68 ± 5.28	4.75 ± 3.42
I090616	− 7.53 ± 0.83**	− 0.19 ± 0.23*	+ 2.07 ± 1.23	+ 0.05 ± 0.08*	+ 6.36 ± 5.77	+ 8.51 ± 3.74*
I090521	− 7.79 ± 2.42	− 0.26 ± 0.01	+ 0.16 ± 3.61	+ 0.03 ± 0.08	+ 19.6 ± 5.04 **	− 1.10 ± 3.26
I070593	− 5.90 ± 2.42	− 0.18 ± 0.36	− 2.57 ± 3.61	− 0.03 ± 0.10	− 6.60 ± 5.67	− 1.68 ± 3.67
I071026	− 7.36 ± 0.00	− 0.23 ± 0.27	− 2.52 ± 3.61	− 0.07 ± 0.15	− 9.06 ± 0.06	− 3.73 ± 4.80
LMR	+ 66.4 ± 2.42	+ 1.36 ± 0.10	+ 4.90 ± 3.21	+ 0.07 ± 0.01	− 1.95 ± 2.77	− 0.94 ± 2.15
Heritability (H^2^)	0.90	0.64	0.08	0.08	0.04	0.01
Location variance (L)	74.22	16.12	15.48	146.7	28.5	5.80
Genotype variance (G)	473**	0.33**	149	0.02	70.0	92.0
G × L variance	35,106**	5.32*	2244.6**	0.00	2347	533.6
Residual variance	59.20	4.77	145.7	3.72	1997.2	882.46
Grand mean	7.70	1.27	7.64	1.17	26.53	10.32
SE	0.9	0.03	0.7	0.02	0.61	0.37
Minimum	− 8.46	− 0.26	− 4.43	− 0.07	− 9.06	− 3.73
Maximum	65.6	1.36	4.90	0.05	19.60	8.51
SD	21.38	0.58	16.47	0.42	51.7	31.33
CV (%)	278.1	45.8	215.8	35.73	194.9	303.7
n Replicates	3	3	3	3	3	3
n Locations	8	8	8	8	8	8
n Genotypes	12	12	12	12	12	12

*P < 0.05, **P < 0.01. The statistics listed for every trait are broad-sense heritability, genotype variance, residual variance, grand mean, SD = Standard Deviation, the coefficient of variation (CV %), the number of replications (n Replicates), and the number of genotypes (n Genotypes). The statistics shown are the estimates derived.

**Figure 2:**
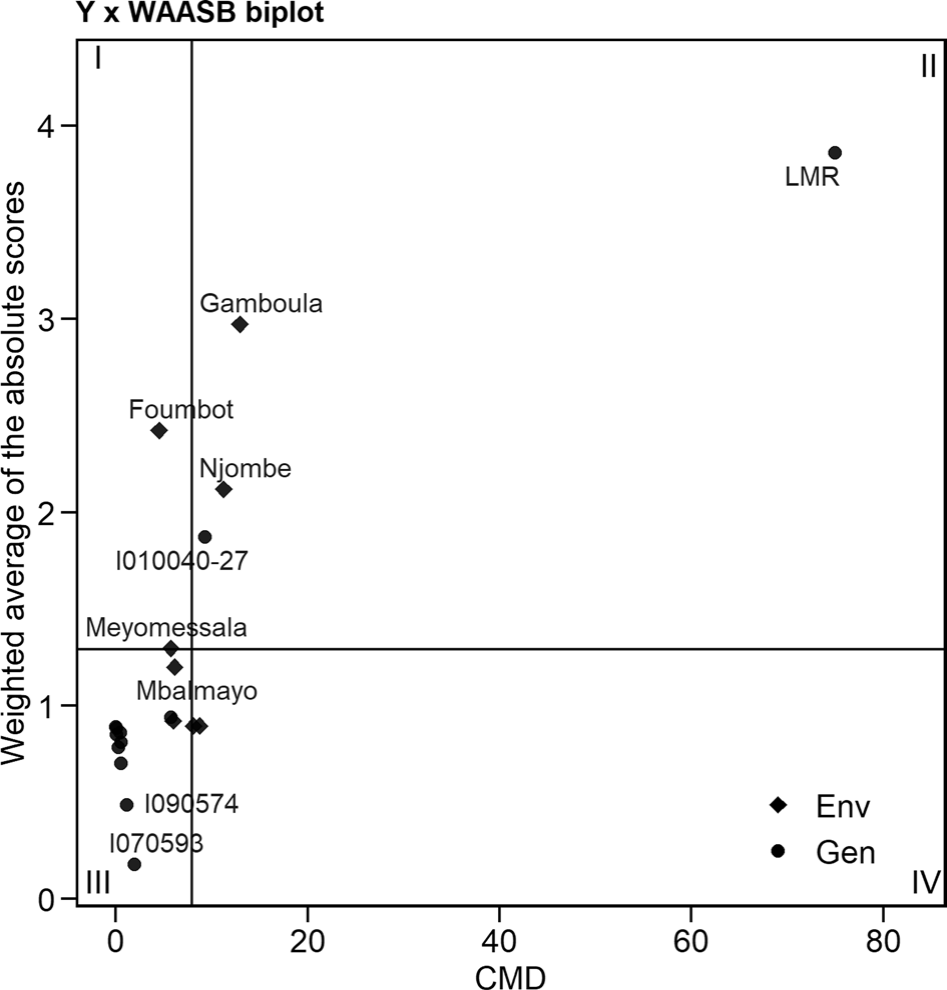
Genotype and genotype by environment interaction (GGE) biplot of the CMD Incidence versus a weighted average of absolute scores for the best linear unbiased predictions of the genotype versus environment interaction (WAASB) of 12 genotypes evaluated in eight environments (combination of 2 cultivation years). (R V3.6.2, https://cran.r-project.org/web/packages/metan/).

The quadrants in Fig. 2 represent the four classes of cassava genotypes/location for a joint interpretation of CMD performance and stability, using the weighted average of absolute scores for the BLUP of the genotypes and location interaction (WAABS) in the eight locations. The 1st quadrant shows that no genotype contributed much to the genotype and location interaction, but there were 2 locations—Foumbot and Meyomessala—that displayed a high discriminative ability for CMD. The 2nd quadrant includes two genotypes, I010040-27 and LMR, which were the most infected and unstable. The two locations included on that quadrant (Gamboula and Njombe), in addition to high disease incidence, also had good discrimination ability for the genotypes. Ten genotypes fell in the 3rd quadrant and are considered as low-infection genotypes, because of the low WAABS values for CMD. No location was included in 3rd quadrant, which was the environment with low infection and discrimination ability. There were no broadly adapted genotypes to be displayed in the 4th quadrant; however, there were 2 locations (Ekona and Meiganga) that had high CMD incidence but with low discriminative ability.

For CAD, the BLUP breeding values ranged from − 4.43 ± 2.61 for improved genotypes (I070557), which showed the lowest CAD incidence,—to + 4.90 ± 3.21 for LMR which also had the highest CAD severity (+ 0.07 ± 0.01) (Table 2). Six genotypes had CAD incidence above the grand mean, but the predicted disease incidence was below 15% for all genotypes (Fig. 1B).

Results from all genomic predictions showed higher heritability for CMD incidence (0.90) and severity (0.64) compared with CAD. The linear mixed model test indicated highly significant effects (*p* < 0.001) in genotype x environment interactions for all the parameters (Table 2).

### Best linear unbiased predictors (BLUPs) for the pests: WF and CGM

The predicted values for whiteflies ranged from − 9.06 ± 0.06 on genotype I071026 to + 19.60 ± 5.04 on I090521, which had the highest number of individuals per plant. The highest BLUP for CGM (+ 8.51 ± 3.74) was predicted on I090616, while the lowest was on I071026 (− 3.73 ± 4.80) (Table 2).

### BLUP for fresh root yields, biomass, dry matter, and total carotenoids content (TCC)

**Figure 3:**
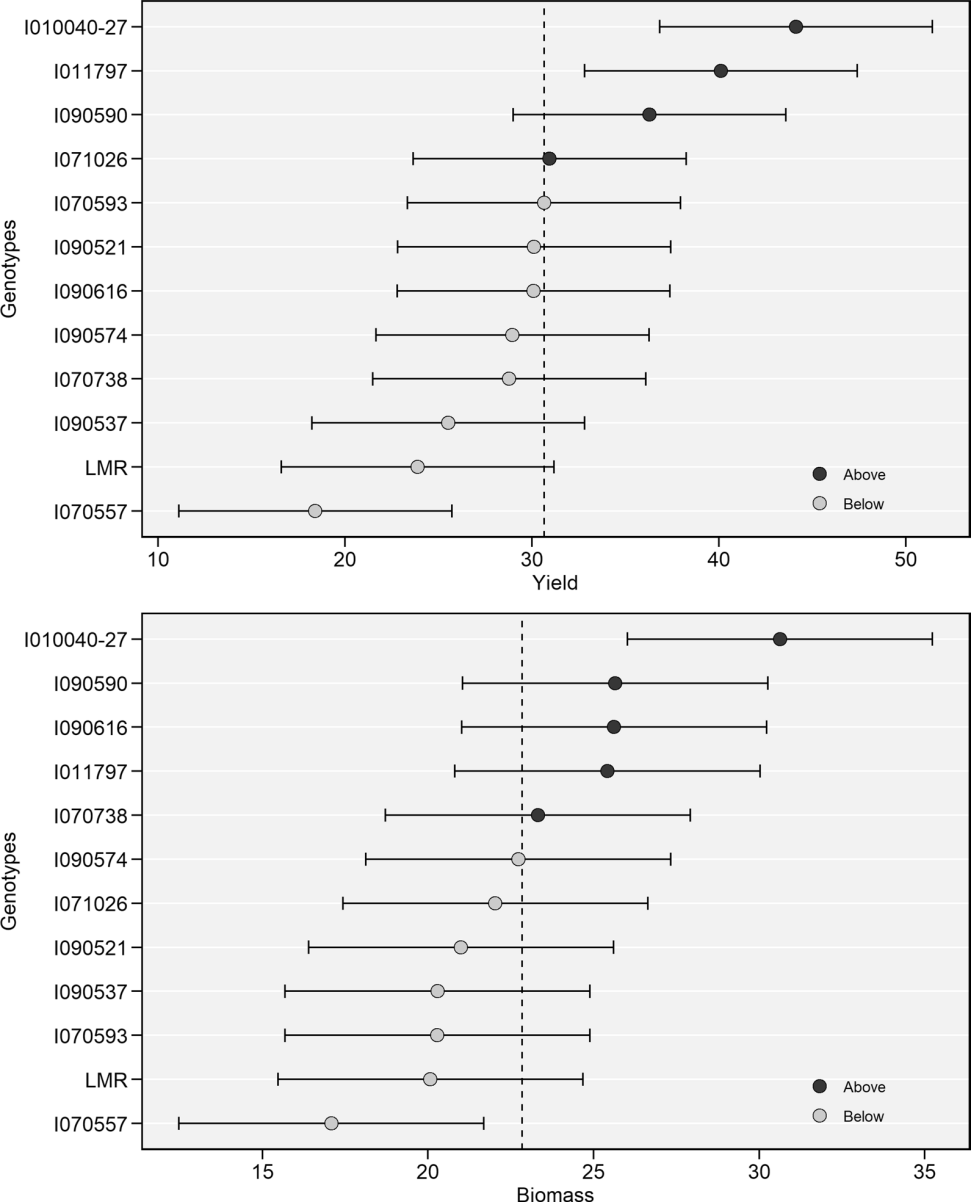
Best linear unbiased prediction for biomass (**A**) and fresh root yield (**B**) for 12 genotypes. Black and grey circles represent the genotypes that had BLUP above and below of BLUP mean, respectively. Horizontal error bars represent the 95% confidence interval of a prediction considering a two-tailed *t* test. (R V3.6.2, https://cran.r-project.org/web/packages/metan/).

Two genotypes (I010040-27 and I011797) stood out for having the highest predicted fresh root yield means among all the tested genotypes (Table 3). The yields of genotypes I090590 and I0701026 were also above the grand mean (Fig. 3B). The predicted breeding values for fresh root yield ranged from − 12.2 ± 2.23 for genotype I070557, which showed the lowest root yield, to + 13.5 ± 1.89 for genotype I010040-27, which had the highest root yield. For cassava above-ground biomass, which included stems and leaves, the predicted breeding values ranged from − 3.73 ± 1.62 for genotype I071026, which showed the lowest value, to + 8.51 ± 1.57 for genotype I090616, which had the highest aboveground biomass. The biomass of five genotypes was above the grand mean (Fig. 3A) The predicted breeding values for dry matter ranged from − 4.03 ± 0.02 for genotype I070593, which showed the lowest DM content, to + 9.36 ± 3.03 for the local landrace LMR, which had the highest DM content (Table 3). Predicted total carotenoid content (TCC) was the highest (+ 5.04 ± 0.17) for improved genotype I070593 compared with LMR, which showed the lowest (− 3.90 ± 0.06%) (Table 3).

**Table 3:** Best linear unbiased predictors (BLUPs) of breeding values with standard errors for fresh root yield (FRY), biomass yield (BY), dry matter (DM), total carotenoid content (TCC) of cassava genotypes across eight locations.

Genotype/statistics	FRY	BY	DM	TCC
I070593	− 0.01 ± 1.91	− 1.68 ± 1.59*	− 4.03 ± 0.02	+ 5.04 ± 0.17**
I071026	+ 0.29 ± 1.95	− 3.73 ± 1.62	− 2.96 ± 0.03	+ 1.95 ± 0.16**
I070557	− 12.2 ± 2.23**	− 0.25 ± 0.62**	− 3.19 ± 0.02	+ 1.09 ± 0.17
I070738	− 1.87 ± 1.91	− 2.85 ± 1.59	− 4.01 ± 0.03	+ 0.43 ± 0.17*
I011797	+ 9.45 ± 1.91**	− 2.28 ± 1.59*	+ 0.63 ± 0.02	− 2.06 ± 0.22**
I010040-27	+ 13.5 ± 1.89**	+ 1.26 ± 0.57**	− 1.19 ± 0.01	− 2.56 ± 0.17**
LMR	− 6.78 ± 0.42	− 0.94 ± 1.63	+ 9.36 ± 3.03	− 3.90 ± 0.06
I090590	+ 5.64 ± 1.93*	− 0.73 ± 0.59*	− 1.08 ± 0.01	–
I090537	− 5.14 ± 1.99*	− 0.93 ± 0.65	− 0.32 ± 0.00	–
I090574	− 1.70 ± 1.91	+ 4.75 ± 0.22	+ 3.26 ± 1.01	–
I090616	− 0.57 ± 1.89	+ 8.51 ± 1.57	− 0.49 ± 0.01	–
I090521	− 0.54 ± 1.95	− 1.10 ± 1.62	+ 3.44 ± 1.02	–
Heritability (H^2^)	0.24	0.14	0.58	0.60
Location variance (L)	237.4*	113.8*	42.2	13.03
Genotype variance (G)	57.39**	16.07**	0.00	723.74**
Gen × Loc variance	56.01**	13.25**	0.61	28.52**
Residual variance	124.0	84.52	9.28	38.00
Grand mean	30.66	22.84	38.33	5.51
SE	0.79	0.57	3.85	0.06
Minimum	− 12.20	− 8.29	− 3.73	− 3.90
Maximum	13.5	10.63	8.51	5.04
SD	16.54	11.94	35.66	2.56
CV (%)	54	52.33	93.56	40.76
n Replicates	3	3	3	3
n Locations	8	8	8	8
n Genotypes	12	12	12	6

*P < 0.05, **P < 0.01. The statistics listed for every trait are broad-sense heritability, genotype variance, residual variance, grand mean, SD = Standard Deviation, the coefficient of variation (CV %)), the number of replications (n Replicates). The statistics shown are the estimates derived.

Overall, the highest heritability (0.60) among the traits was estimated from TCC (Table 3). The linear mixed model indicated highly significant effects (*p* < 0.001) for genotype and environmental interactions for all tested traits (Table 3).

**Figure 4:**
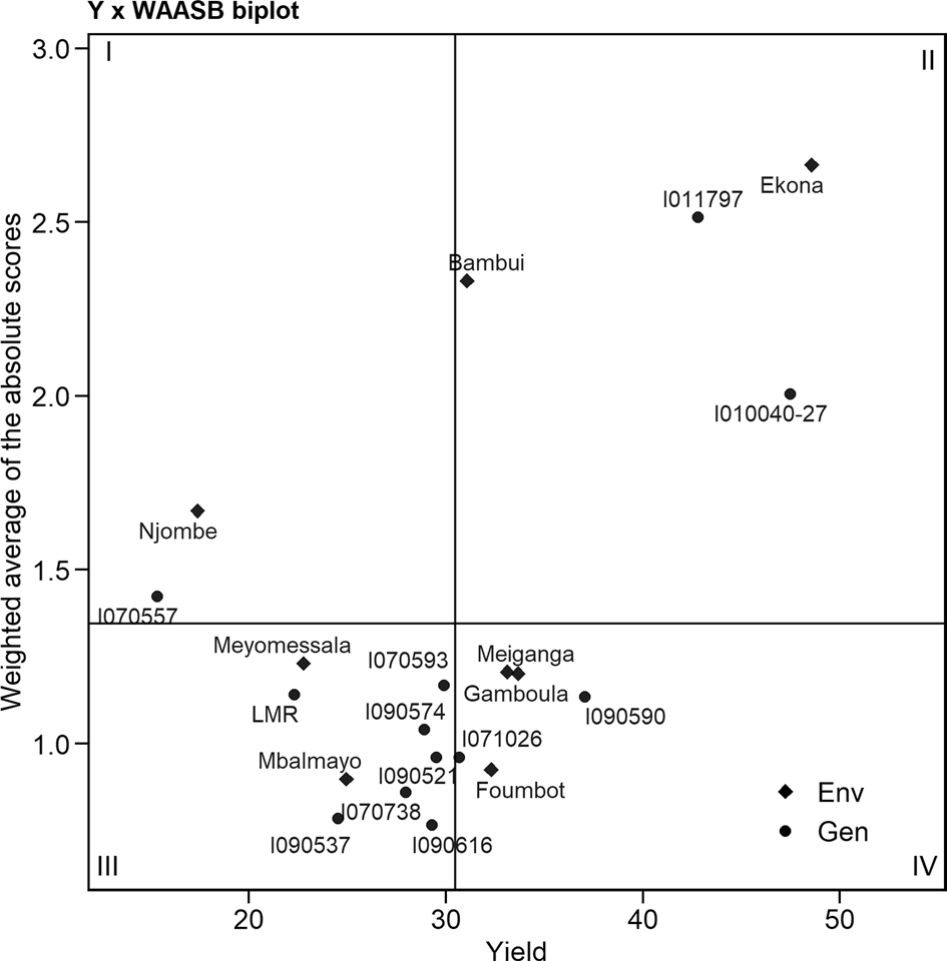
Biplot of the fresh root yield versus a weighted average of absolute scores for the best linear unbiased prediction s of the genotype versus environment interaction (WAASB) of 12 genotypes evaluated in eight environments. A hypothetical highly productive and broadly adapted genotype is depicted by a black circle. (R V3.6.2, https://cran.r-project.org/web/packages/metan/).

The quadrants in Fig. 4 represent the 4 classes of cassava genotypes/location for a joint interpretation of fresh root yield and stability using the weighted average of absolute scores for the BLUP of the genotype and location interaction (WAABS) in the 8 locations. The 1st quadrant shows that genotype I070557 was the most unstable and contributed much to the genotype and location interaction. The Njombe environment, which is displayed in that quadrant, had a high discriminative ability for fresh root yield. The 2nd quadrant includes two genotypes I010040-27 and I011797 which are the most productive but unstable genotypes. The two locations included on that quadrant, Ekona and Bambui, in addition to providing high performance, also provided good discrimination ability for the genotypes. Six genotypes fall in the third quadrant and are considered as low productive genotypes because of the low WAABS values. The Mbalmayo and Meyomessala environments included in this quadrat had low production and low discrimination ability. Two genotypes I090590 and I071026 were broadly adapted and are displayed in the fourth quadrant. Three locations in this quadrant, Foumbot, Gamboula, and Meiganga, had high yield but with low discriminative ability.

## Discussion

Multi-environment experiments are a primary focus in plant breeding programs; therefore, their prediction accuracy, compared with observed value, is crucial for selection recommendation of cultivars, and the identification of mega-environments. This study showed variable responses of a set of new cassava genotypes for food and nutritional value in contrasting environments in Cameroon, thus justifying the necessity to evaluate genotype by environment interactions. This study, together with the previous study conducted in the same locations, but with different genotypes^8^, provide a highly important set of data on the effects of genotype and environment on 28 cassava genotypes that are in advanced stages of development. Moreover, the two studies together, are a rarity in evaluations that covers a very wide spectrum of pest/disease resistance, yield, and nutritional value, all with a significant potential contribution to food and nutritional security in Central Africa. The four agroecological zones in which the genotypes were tested are representative of the agro-ecologies of the Congo Basin, including nearly all states in Central Africa, except the Sahelian zone^33^. Importantly, we found that the environment was not the main determinant of a genotype response to cassava mosaic virus (CMD) as the heritability was close to one; this opens the possibility of introducing genotypes with good performance in terms of productivity, yield stability, and resistance to pest and disease into areas with similar characteristics as in Cameroon. Indeed, as with varieties for industrial processing, the tested genotypes were initially selected based on their resistance to CMD and yield, as well as provitamin A content for the yellow root genotypes, traits in which variability comes from genetic differences, with very little contribution from environmental factors^34^. We noted, however, two genotypes, I070593 and LMR, that had CMD severity scores of 2.8 and 2.6 respectively on average for both years, although the incidence on the same genotypes was nearly nil (less than 2% on average). Our two checks, I010040-27 and LMR, displayed on average the same incidence and severity as in the previous study^8^; however, the other improved varieties (10 in total) were five-fold more resistant to CMD compared with the 16 of the previous study, thus confirming the improvement made on the current set of varieties with respect to CMD resistance and the necessity to disseminate those genotypes. The significant interaction observed between environment and genotype for CMD infection could be related to the virus strains present in the locations as previous studies established the presence of various CMD strains in Cameroon, particularly the Ugandan variant which is the most virulent and present in the east region (Gamboula)^35^.

Typical CMD symptoms on cassava plants are misshapen leaves which hamper the growth of the plant and reduce root yields, hence lowering productivity and profitability^21,36^. Contrary to CMD, Cassava Anthracnose Disease (CAD) was found mostly in the forest areas where relative humidity and rainfall significantly influence the levels of CAD inoculum pressure, in addition to favoring the development of its vector *Pseudotheraptus devastans* Dist (Het. Coreidae)^23^. CAD severity was statistically similar on all genotypes, but with low incidence. Genotype I071026 did not show CAD symptoms during the 2nd year. CAD has become an economic threat to cassava production as severe outbreaks have been reported^22^. Affected plants display necrotic lesions on leaves and stem, reducing planting material availability^37^. Careful selection of clean planting material and good field maintenance (timely weeding and field aeration) could help in reducing CAD pressure in the forest zone where it is most prevalent.

Interestingly, the genotype I090521 did not show any CMD symptoms over the two trial years despite harboring the highest number of whiteflies per plant as previously demonstrated with four genotypes (01/0098, 01/1086-55, 95/0211, and 98/0581)^8^; however, the lack of CMD symptoms could mean that whiteflies did not carry enough virus loads to efficiently transmit the virus, as an increase in whitefly numbers can lead to the higher efficiency of CMD transmission on cassava^38^ and also could likely be explained by the resistance mechanism of this cassava line. This contradicts other findings^39^ which reported that 10 whiteflies per plant constituted an adequate population for the spread of CMD due to their persistent mode of transmission.

The observed resistance to major disease was translated into a higher yield for the improved genotypes.These varieties could also be carriers of genes for higher yield. Current cassava production in Cameroon (and in the Central Africa region respectively) stands at 5,798,909 tons (resp. 52,019,756 tons) with an average yield of 14.5 t/ha (resp. 8.9 t/ha)^3^. Therefore, promoting the new genotypes with an average yield of 31 t/ha could potentially increase production in Cameroon to 12,353,035 tons with the current production areas (398,485 ha) which would represent an increase from 11 to 24% of the cassava production in Central Africa. It is worth noting the sharp increase in yield of the check I010040-27 which almost doubled within two years after its first evaluation. Cassava's potential to alleviate food and nutritional security in Central Africa would substantially increase if neighboring countries sharing similar ecologies with Cameroon (Gabon, Equatorial Guinea, Central African Republic, Congo)—where cassava is an essential food security crop—also adopt the new genotypes.

Two genotypes (I090590, and I0701026) produced the highest and most stable fresh root yield across locations, which are qualities that favors them for dissemination throughout the targeted environments to improve cassava yields and hence food security. Overall, tested cassava genotypes performed best at Ekona and Bambui, probably because of the high K and Organic C in their soils^40^. Dry matter content was constant for all genotypes across all environments. As a polygenic trait, dry matter varies from one genotype to another (20–40%) and is usually stable across locations^41^. Genotypes with high dry matter (> 30%) are generally mealy with high starch content^42–44^, making them suitable for processing into flour and starch which have been shown to improve the potential for cassava adoption and commercialization for income generation and livelihoods improvement^15,45^.

Nutritionally-biofortified genotypes evaluated in this study contain up to 6-folds higher provitamin A β-carotene compared with the local genotype, underlining the potential contribution of the biofortified genotypes to nutritional security in Cameroon and central Africa. These varieties have almost 3 times (up to 11.1 μg/g) the level of carotenoids contents of our previous set of yellow varieties^8^, thus justifying the improvement of their nutritional content and the need to promote them for nutritional security in central Africa. Total carotenoid content is known to be affected by the location in which the genotype is grown, especially in sweet potato (*Ipomoea batatas* (L.)^46^, but it appears not to be the case for cassava as the levels of provitamin A (β-carotene) of a genotype were stable across all the different environments used in the present study and elsewhere^47^. These results are very encouraging, but it would be necessary to conserve as much of the provitamin A as possible when cassava is transformed into different products—including cooking—to harness the full potential of biofortified cassava’s contribution to reductions in vitamin A deficiency^48^.

## Conclusion

This study identified high-yielding cassava genotypes with higher levels of resistance/tolerance to pests and diseases and with elevated provitamin A-improved nutritional content. Disseminating the selected genotypes for cassava production in Cameroon—and by extension elsewhere in Central Africa—would improve cassava production for food and nutritional security. Despite the good performance of most of the tested genotypes, there is also a strong need to conduct consumer preference studies to match the usability of these newly developed varieties to actual use of cassava in the area of introduction to ensure proper adoption by the end-users in the various agro-ecologies.

## Materials and methods

### Cassava genotypes

IITA had the permission to collect all the plants used in this study. The improved cassava genotypes used were selected in Nigeria by the IITA cassava breeding unit and sent to IITA Cameroon. Accession names and their pedigree are available in the cassava database (Table 4) (www.cassavabase.org). All improved genotypes were tested along with two cassava genotypes from our recent work^8^ which were used here as references (1) for high yield (I010040-27) and (2) for high susceptibility to cassava mosaic virus disease (LMR). Including reference genotypes is a common practice in such studies. The present and previous^8^ study were conducted in the same locations (not same fields) in Cameroon and followed a similar methodology to provide a basis for comparisons between them. The first study^8^ used mostly white-fleshed genotypes oriented toward industrial processing, while the second (present) study emphasized a new set of white-fleshed genotypes for boiling and yellow-fleshed genotypes with higher total carotenoids content. The studies complied with the local and national regulations in Cameroon.

**Table 4:** Cassava genotypes used in the trial.

Accession	Cassava base name	Pedigree	Flesh color
I070593	IITA-TMS-IBA070593	IITA-TMS-IBA011277/IITA-TMS-IBA990067	Yellow
I010040-27	IITA-TMS-IBA010040	IITA-TMS-IBA010040/?*	Yellow
I011797	IITA-TMS-IBA011797	IITA-TMS-IBA950379/TMEB117	Yellow
I070557	IITA-TMS-IBA070557	IITA-TMS-IBA011663/IITA-TMS-IBA940330	Yellow
I070738	IITA-TMS-IBA070738	IITA-TMS-IBA011649/IITA-TMS-IBA051237	Yellow
I071026	IITA-TMS-IBA071026	IITA-TMS-IBA011277/IITA-TMS-IBA011412 (4X)	Yellow
I090521	IITA-TMS-IBA090521	IITA-TMS-IBA974763/MAUNJILI	White
I090537	IITA-TMS-IBA090537	IITA-TMS-IBA961569/IITA-TMS-IBA961569	White
I090574	IITA-TMS-IBA090574	IITA-TMS-IBA961632/CM5306-8	White
I090590	IITA-TMS-IBA090590	IITA-TMS-IBA972205/MCOL 1468	White
I090616	IITA-TMS-IBA090616	IITA-TMS-MOK980068/CM6921-3	White
LMR	LMR	NA/NA	White

*/? Denote half sibling.

### Trial locations and characteristics

**Figure 5:**
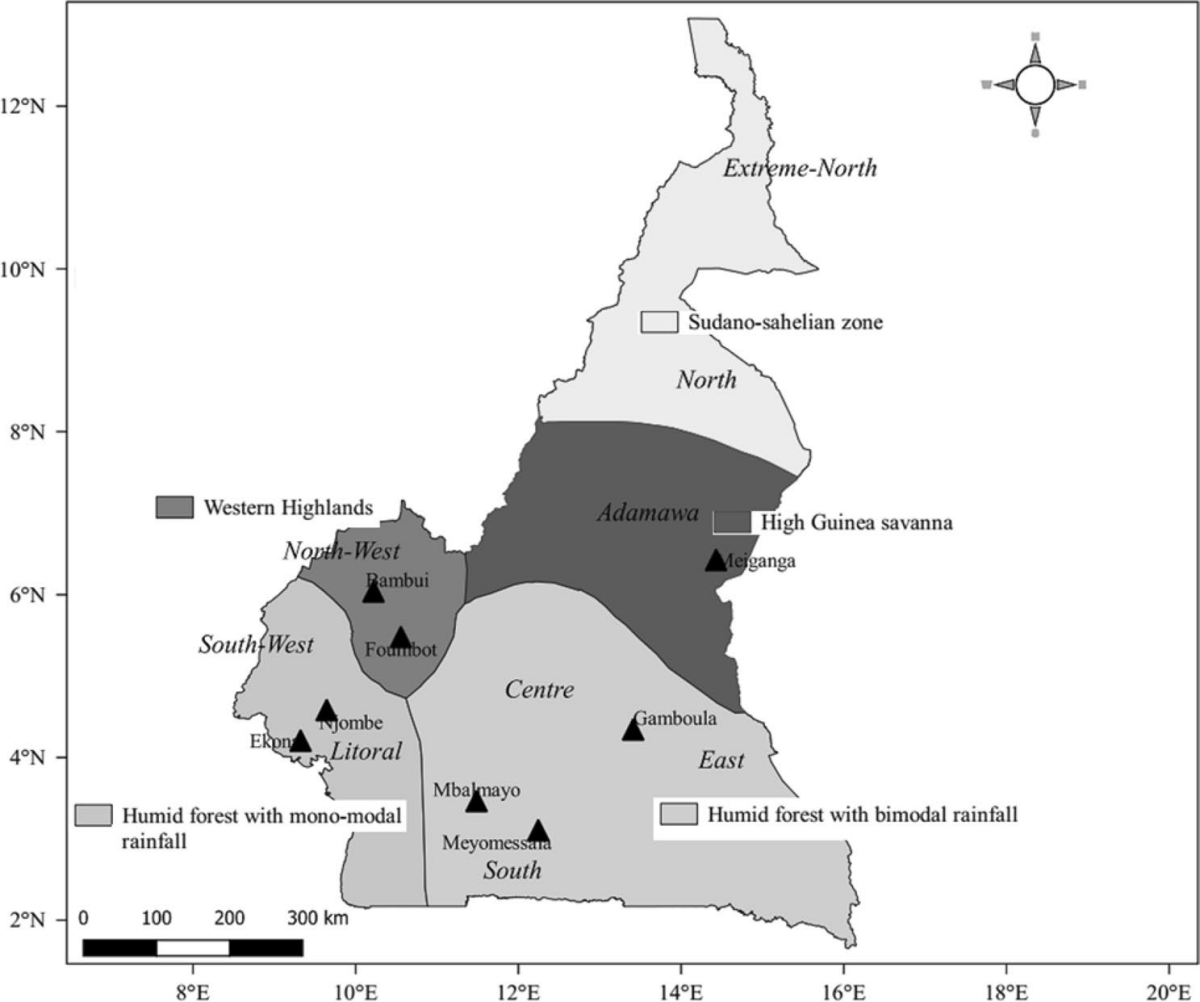
Locations of the cassava multilocation trial (Arcgis V10.3.1, https://desktop.arcgis.com/en/arcmap/).

Field trials were established in eight locations during the 2016/2017 and 2017/2018 first cropping season that typically starts in March of each year. These locations are grouped into 4 agroecological zones as classified by the Institute Agronomic Research for Development of Cameroon (IRAD). The locations of Mbalmayo, Meyomessala, and Gamboula are in the humid forest zone which is characterized by a bimodal rainfall pattern, while the locations of Njombe and Ekona are in the humid forest with monomodal rainfall patterns. The locations of Foumbot and Bambui are in the Western Highlands while Meiganga is in the high Guinean savannah (Fig. 5). Vegetation in these locations as representative of the land cover of other countries of Central Africa^33^.

### Trial set-up and monitoring

The trial was set-up in a completely randomized block design with three replicate blocks comprised of 5 × 6 m plots of 42 plants (with 1 m spacing between and within rows) of each of the 12 genotypes. Plants were grown under rainfed conditions for 12 months and managed according to prevailing farmer practices. Pesticides and fertilizers were not used at any time during the trials. Plots were manually weeded with machete and hoe as needed.

### Soil sampling

At each location, 5 soil samples were collected from each plot and mixed thoroughly in a plastic basin to obtain a composite soil sample. A subsample of 250 g of soil from each plot was used for the determination of various soil physical and chemical properties at the IITA-Cameroon Analytical Laboratory. Soil samples were air-dried and ground to pass through a 2-mm sieve. The sample was further ground to pass through a 0.5 mm sieve for C and N analysis. Soil pH in water was determined in a 1:2.5 (w/v) soil: water suspension. Organic C was determined by chromic acid digestion and spectrophotometric analysis^49^. Total N was determined from a wet acid digest^50^ and analyzed by colorimetric analysis. Exchangeable Ca, Mg, K, and P were extracted using the Mehlich-3 procedure^51^, and the cations were determined by flame atomic absorption spectrophotometry. Exchangeable P from the resulting extract above was determined with the molybdate blue procedure^52^. Particle size was determined with a hydrometer.

### Pests and diseases assessment

Cassava disease incidence and severity were evaluated at 3, 6, and 9 months after planting on 10 plants per plot, excluding border rows, while alternating plants within a row. On each plant, the number of whitefly adults (*Bemisia tabaci* Gennadius) was counted on the top (the first five apical leaves of the plant tilting the apex) of the plant, while the number of nymphs was counted on the 14th fully developed leaf^53^. The cassava green mite (CGM) was counted on the fifth fully developed leaf using a head lens (OptiVISOR Optical Glass Binocular Magnifier, 10× ). CMD incidence (presence/absence) was scored on the whole plant foliage, while CAD incidence and severity were scored based on disease symptoms on the stems. Disease and damage scoring was done on a scale of 1 (no symptoms observed) to 5 (severe symptoms on plant parts)^54^.

At harvest (12 months after planting), above-ground biomass (stems and leaves) and storage roots were weighed using Macro scales type with optional accessories (Macro-Line 800050, PRESOLA). Samples of 500 g of the fresh root of each genotype were collected and oven-dried at 60 °C for 48 h to measure their dry matter content. Total carotenoid content (TCC) in storage roots was determined within 24 h of harvest using iCheck Carotene following the BioAnalyt protocol^53^. Only yellow flesh genotypes were considered for the carotenoids content analysis as the yellow coloration of the parenchyma is closely related to carotenoid content^55^. The local landrace LMR was included as a check.

### Statistical analysis

Incidence data was calculated for CMD and CAD for each plot by calculating the percentage of sampled plants that showed symptoms of the diseases.

We used the metan package of the R 3.6.2 software^56^ to perform a stability analysis of multi-environment trial data (MET^57^) using parametric and non-parametric. MET allows the identification of genotypes that display a small temporal variability—which is desired by breeders and is beneficial to growers, or cultivars that are consistent from location to location—which is desired by and is beneficial to seed companies and breeders^57^. Incidence data were calculated for CMD and CAD by calculating the percentage of sampled plants that showed symptoms of the diseases. Whitefly and CGM densities were log-transformed to reduce heteroscedasticity inherent in insect and mite counts. The best linear unbiased prediction (BLUP) was used to predict Breeding Values (BV) of each genotype for cassava mosaic disease incidence and severity, cassava anthracnose disease incidence and severity, whitefly and cassava green mite densities, fresh root yield (FRY), biomass (BY), dry matter (DM), total carotenoid content (TCC), CMD and CAD severity. The BLUP was performed using the linear mixed model's approach (ANOVA) that considered cassava genotype as fixed factor and locations and year as random factors. The model also included genotype by location (G × L) interactions.

To select genotypes that combine high performance and stability, we introduced the weighted average of absolute scores from the singular value decomposition of the matrix of best linear unbiased predictions for the genotype × environment interaction effects generated by a linear mixed effect model index (WAASB), which is a superiority index that allows weighting between performance (in our study, fresh root yield and CMD incidence) and stability (WAASB index). The first step is rescaling FRY and WAASB; CMD and WAASB that can be directly used to compare genotypes. The best values for FRY and CMD are the highest value and for WAASB is the lowest value^58–60^.

## Data Availability

The datasets generated during and/or analyzed during the current study are available from the corresponding author on reasonable request.
